# The harsh microenvironment in infarcted heart accelerates transplanted bone marrow mesenchymal stem cells injury: the role of injured cardiomyocytes-derived exosomes

**DOI:** 10.1038/s41419-018-0392-5

**Published:** 2018-03-02

**Authors:** Ming Hu, Guixian Guo, Qiang Huang, Chuanfang Cheng, Ruqin Xu, Aiqun Li, Ningning Liu, Shiming Liu

**Affiliations:** Guangzhou Institute of Cardiovascular Disease, the Second Affiliated Hospital of Guangzhou Medical University, Guangzhou, 510260 Guangdong China

## Abstract

Stem cell therapy can be used to repair and regenerate damaged hearts tissue; nevertheless, the low survival rate of transplanted cells limits their therapeutic efficacy. Recently, it has been proposed that exosomes regulate multiple cellular processes by mediating cell survival and communication among cells. The following study investigates whether injured cardiomyocytes-derived exosomes (cardiac exosomes) affect the survival of transplanted bone marrow mesenchymal stem cells (BMSCs) in infarcted heart. To mimic the harsh microenvironment in infarcted heart that the cardiomyocytes or transplanted BMSCs encounter in vivo, cardiomyocytes conditioned medium and cardiac exosomes collected from H_2_O_2_-treated cardiomyocytes culture medium were cultured with BMSCs under oxidative stress in vitro. Cardiomyocytes conditioned medium and cardiac exosomes significantly accelerated the injury of BMSCs induced by H_2_O_2_; increased cleaved caspase-3/caspase-3 and apoptotic percentage, and decreased the ratio of Bcl-2/Bax and cell viability in those cells. Next, we explored the role of cardiac exosomes in the survival of transplanted BMSCs in vivo by constructing a Rab27a knockout (KO) mice model by a transcription activator-like effector nuclease (TALEN) genome-editing technique; Rab27a is a family of GTPases, which has critical role in secretion of exosomes. Male mouse GFP-modified BMSCs were implanted into the viable myocardium bordering the infarction in Rab27a KO and wild-type female mice. The obtained results showed that the transplanted BMSCs survival in infarcted heart was increased in Rab27a KO mice by the higher level of Y-chromosome Sry DNA, GFP mRNA, and the GFP fluorescence signal intensity. To sum up, these findings revealed that the injured cardiomyocytes-derived exosomes accelerate transplanted BMSCs injury in infarcted heart, thus highlighting a new mechanism underlying the survival of transplanted cells after myocardial infarction.

## Introduction

Stem cell-based therapy for myocardial infarction (MI) has received unprecedented attention over the last decades^1,2^. Bone marrow mesenchymal stem cells (BMSCs), because of their unique properties for easily obtain, multilineage potential, high proliferation, and immune privilege, have become an attractive cell for transplantation therapy to MI^3,4^. Nevertheless, the poor cell survival in the harsh ischemic heart microenvironment limits their therapeutic efficacy, thus urging the identification of new and effective approaches, as well as exploration of mechanisms underlying BMSCs in MI^5^. So far, several approaches have been proposed to improve the survival of engrafted cells, including preconditioning, genetic modification, and improving host tissue environment^6–10^. Many cell types interact in a high coordinated manner to control heart integrity and homeostasis, including cardiomyocytes (CMs), myofibroblasts, immune cells, cardiac-derived stem cells, and endothelial cytes^11,12^. Recently, exosomes have shown to regulate multiple processes, including cell survival, angiogenesis, and immune responses, by mediating the communication among cells/organs^13^. Although CMs do not act as typical secretory cells, exosomes can be secreted from these cells in an inducible manner. Together with trophic factors and signaling molecules, the exosomes secreted from CMs have been proposed to be critical for myocardium by mediating intercellular contacts^14^. It remains largely unknown whether the injured CMs-derived exosomes (cardiac exosomes) have an ability to affect the survival of transplanted BMSCs after MI.

Exosomes are a subfamily of extracellular vesicles (EVs) that correspond to the internal vesicles present in multivescular endosomes (MVEs), and their size usually ranges from 40 to 200 nm^12^. Upon MVEs fusing with plasma membrane, exosomes are constitutively released into the extracellular environment. Rab proteins, a family of GTPases, functionally participate in different steps of intracellular membrane trafficking, including endocytic and secretory processes, as well as exosome production or secretion^15^. Knockdown of Rab27b is suggested to redistribute the MVEs toward perinuclear region, while late endosome and lysosome compartments get accumulated and enlarged in Rab27a genetic inhibition cells. This suggests that Rab27a is necessary for the docking and fusion of MVEs with the plasma membrane, and is also important in exosomes secretion^16^. In order to explore the role of cardiac exosomes in the survival of transplanted BMSCs in vivo, we constructed a Rab27a KO mice model following the implantation of GFP-modified BMSCs into the viable myocardium bordering the infarction in Rab27a KO female mice. Consequently, the survival of transplanted cells was assessed by the expression of Y-chromosome Sry DNA and GFP mRNA, as well as by detecting GFP fluorescence signal intensity. In this study, in vitro and in vivo assays were carried out to determine the effects of the cardiac exosomes on survival of transplanted BMSCs in infarcted heart.

## Results

### Oxidative stress caused apoptosis of CMs and BMSCs

**Fig. 1:**
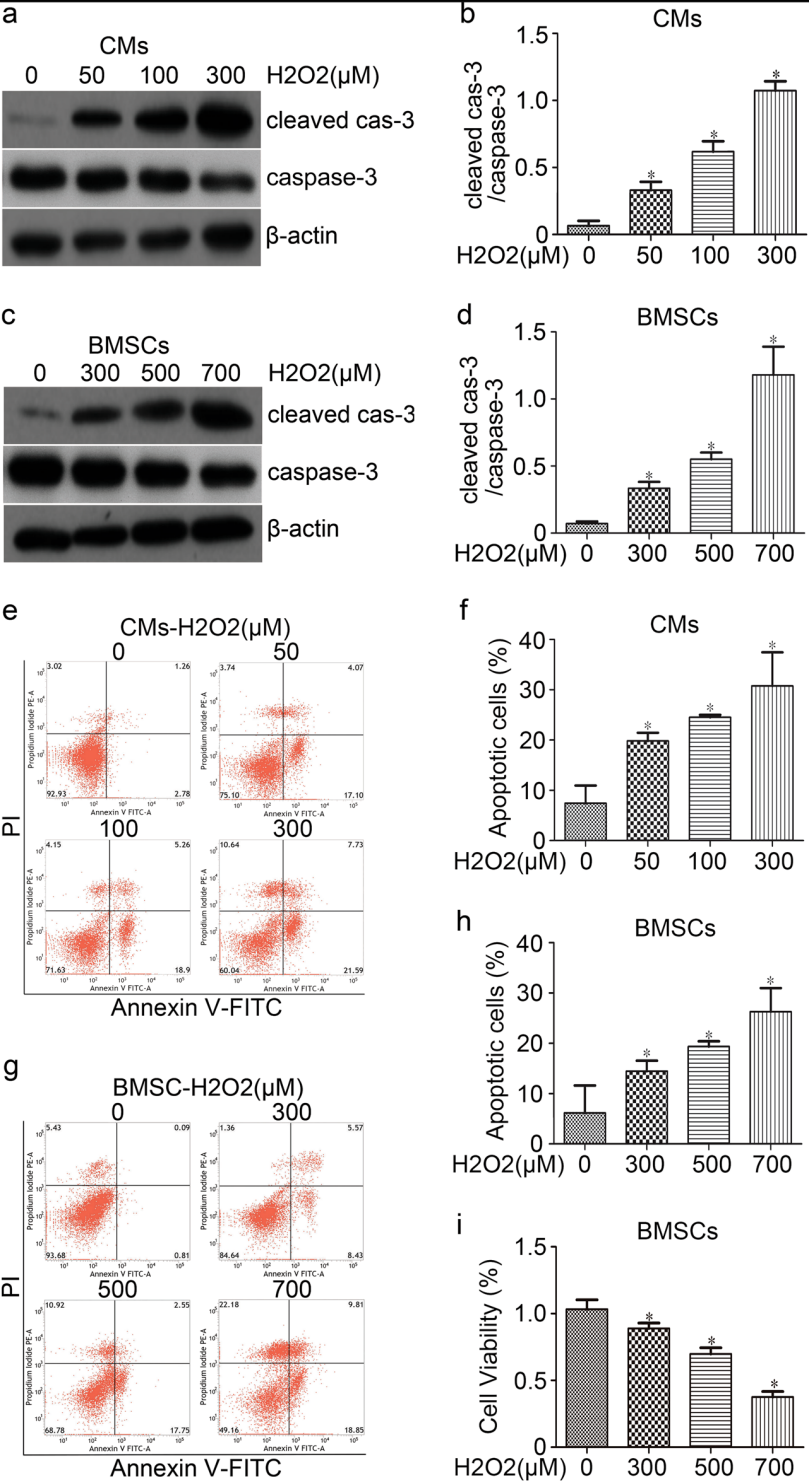
**Oxidative stress injury on CMs and BMSCs in vitro.** H_2_O_2_ dose dependently induced apoptosis in CMs (**a**) or BMSCs (**c**) 24 h post- treatment. Protein lysates were subject to western blot analysis using antibodies as mentioned above. β-actin was employed as a loading control, and quantitative data of cleaved caspase 3 band densities were shown (**b** and **d**). CMs (**e**) and BMSCs (**g**) cell apoptosis were assayed using Annexin V-FITC/PI dual staining by flow cytometry; representative flow images were shown. The percentages of apoptotic CMs and BMSCs cells were summarized as (**f** and **h**). **i** The BMSCs cell viability after treatment with different concentrations of H_2_O_2_ was detected by CCK-8 assay. *p < 0.05 versus control group. All values were expressed as mean ± SD, *n* = 3 for each group

To mimic the oxidative stress microenvironment after MI in vivo, the CMs and BMSCs were exposed to different concentrations of H_2_O_2_ for 24 h. Cells were then harvested for protein collection and subjected to western blot analysis. The CMs apoptosis was positively correlated with H_2_O_2_ concentration, as showed by the elevated cleaved caspase-3/caspase-3 expression (Fig. 1a, b); Annexin V-FITC/PI assay showed that H_2_O_2_ dose dependently induced CMs cell apoptosis ratio by 19.9 ± 1.6%, 24.6 ± 0.5%, and 30.8 ± 6.7% compared to the control group (7.4 ± 3.5%) (*p* < 0.05) (Fig. 1e, f). A serious damage of CMs was caused by 300 μM H_2_O_2_, therefore 100 μM was selected as a stimulating concentration. Furthermore, by increasing the concentration of H_2_O_2_, a significant increase in cleaved caspase-3 was observed in BMSCs (Fig. 1c, d); and BMSCs cell apoptosis ratio by 14.5 ± 2.1%, 19.4 ± 1.0%, and 26.3 ± 4.7%, compared to the control group (6.2 ± 5.4%) (*p* < 0.05) (Figures 1g, h). Additionally, decreased BMSC cell viability was observed when treating cells with 300 μM (88.9 ± 4.0%), 500 μM (69.8 ± 4.6%), 700 μM (37.6 ± 4.1%) H_2_O_2_ compared with NC (*p* < 0.05) (Fig. 1i). As the injury of 700 μM H_2_O_2_ showed to be too aggressive in promoting cell damage, the concentration of 500 μM was selected to stimulate BMSCs.

### Cardiomyocytes conditioned medium accelerated apoptosis of BMSCs under oxidative stress

**Fig. 2:**
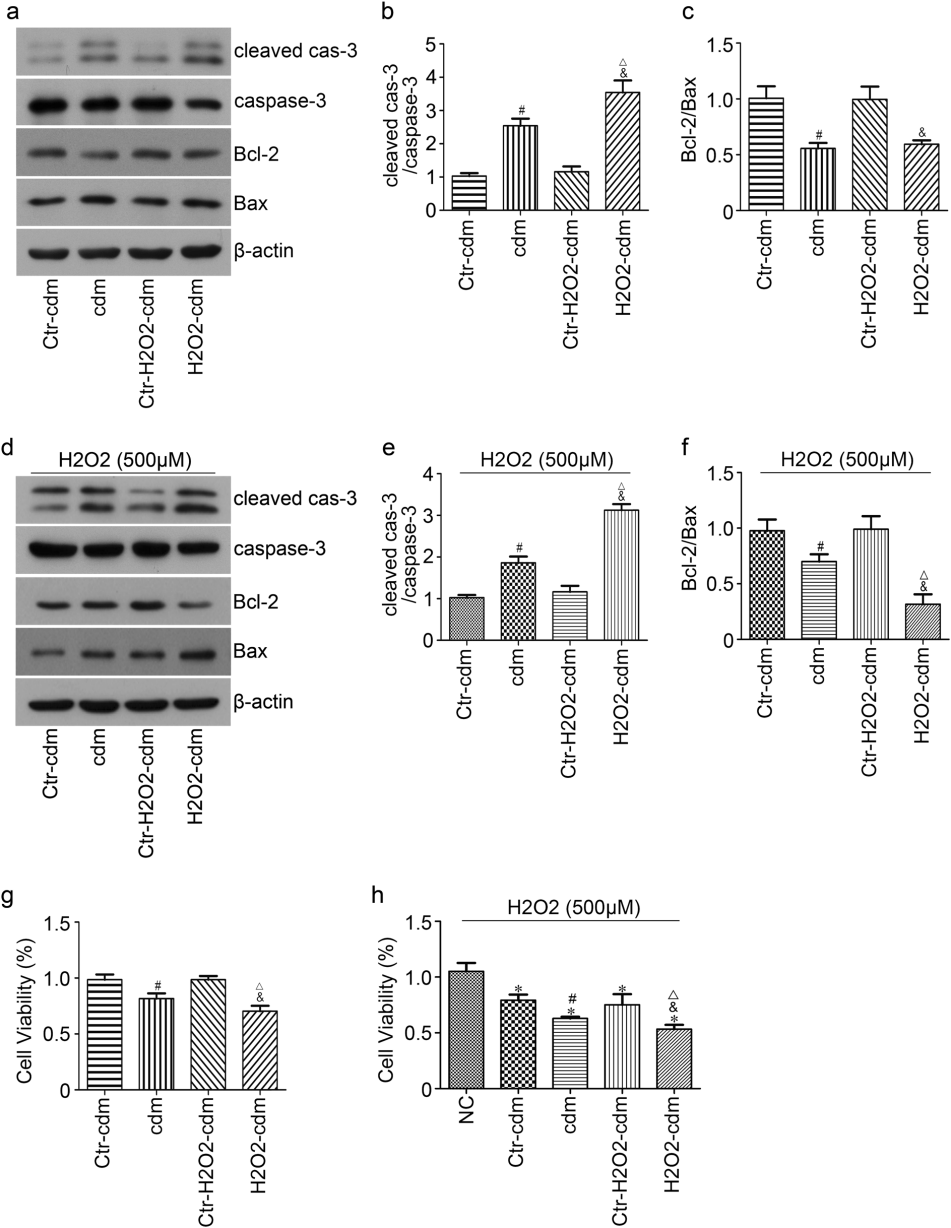
Effects of cardiomyocytes conditioned medium on apoptosis of BMSCs under oxidative stress. **a** BMSCs were lysated for detecting cleaved caspase-3, caspase-3, Bcl-2, Bax after being cultured with cardiomyocytes conditioned medium and treated without (**a**) or with (**d**) 500 μM H_2_O_2_ for 24 h by western blot. Quantitative data of cleaved caspase-3/caspase-3 (**b** and **e**) and Bcl-2/Bax ratio (**c** and **f**) were shown, respectively. The cell viability of BMSCs after being cultured with cardiomyocytes conditioned medium and treated without (**g**) or with (**h**) 500 μM H_2_O_2_ for 24 h was detected by CCK8 assay. *NC* BMSCs cells with DMEM containing 10% FBS, *cdM* serum-free DMEM for culturing CMs for 24 h; *H*_*2*_*O*_*2*_*-cdM* serum-free DMEM, and 100 μM H_2_O_2_ used to treat CMs for 24 h; Ctr-cdM (without CMs) and Ctr-H_2_O_2_-cdM (without CMs) served as control of cdM and H_2_O_2_-cdM, respectively. *p < 0.05 versus NC, ^#^*p* < 0.05 versus Ctr-cdM, ^&^*p* < 0.05 versus Ctr-H_2_O_2_-cdM, ^△^*p* < 0.05 versus cdM. All values were expressed as mean ± SD, *n* = 3 for each group

In order to explore the mechanisms underlying the poor cell survival of implanted BMSCs in the ischemic heart environment, and whether CMs affect the survival of implanted BMSCs by paracrine action, we cultured BMSCs with CMs conditioned medium under 500 μM H_2_O_2_ for 24 h. The protein lysates were prepared and detected by western blot. Briefly, no statistical difference in cleaved caspase-3/caspase-3 expression was found between group Ctr-cdM and Ctr-H_2_O_2_-cdM, whereas it significantly increased in group cdM compared to group Ctr-cdM and in group H_2_O_2_-cdM compared to group Ctr-H_2_O_2_-cdM (Fig. 2d, e). In addition, western blot revealed that group cdM and group H_2_O_2_-cdM exhibited a significantly decreased ratio of Bcl-2/Bax compared to control group (Figure 2f). Moreover, cell viability was greatly decreased in group cdM (62.9 ± 1.6) compared with group Ctr-cdM (79.3 ± 5.0) and in group H_2_O_2_-cdM (54.7 ± 2.1) relative to group Ctr-H_2_O_2_-cdM (75.2 ± 9.6) upon treatment with 500 μM H_2_O_2_ for 24 h (*p* < 0.05) (Figure 2h). Meanwhile, protein expression changed after culturing BMSCs with CMs conditioned medium in normal culture (without 500 μM H_2_O_2_) for 24 h in vitro; increased expression in cleaved caspase- 3/caspase-3 and decreased expression in the ratio of Bcl-2/Bax were found in group cdM relative to group Ctr-cdM and the same changes were found in group H_2_O_2_-cdM compared to group Ctr-H_2_O_2_-cdM (Fig. 2a–c). In addition, decreased cell viability was observed in cdM (81.7 ± 4.6%) compared to H_2_O_2_-cdM (70.5 ± 4.7%) (*p* < 0.05) (Figure 2g). Also, a more serious cell injury was caused by H_2_O_2_-cdM compared to cdM. Accordingly, we explored what substances in the H_2_O_2_-cdM take effects.

### Identification of the cardiac exosomes

**Fig. 3:**
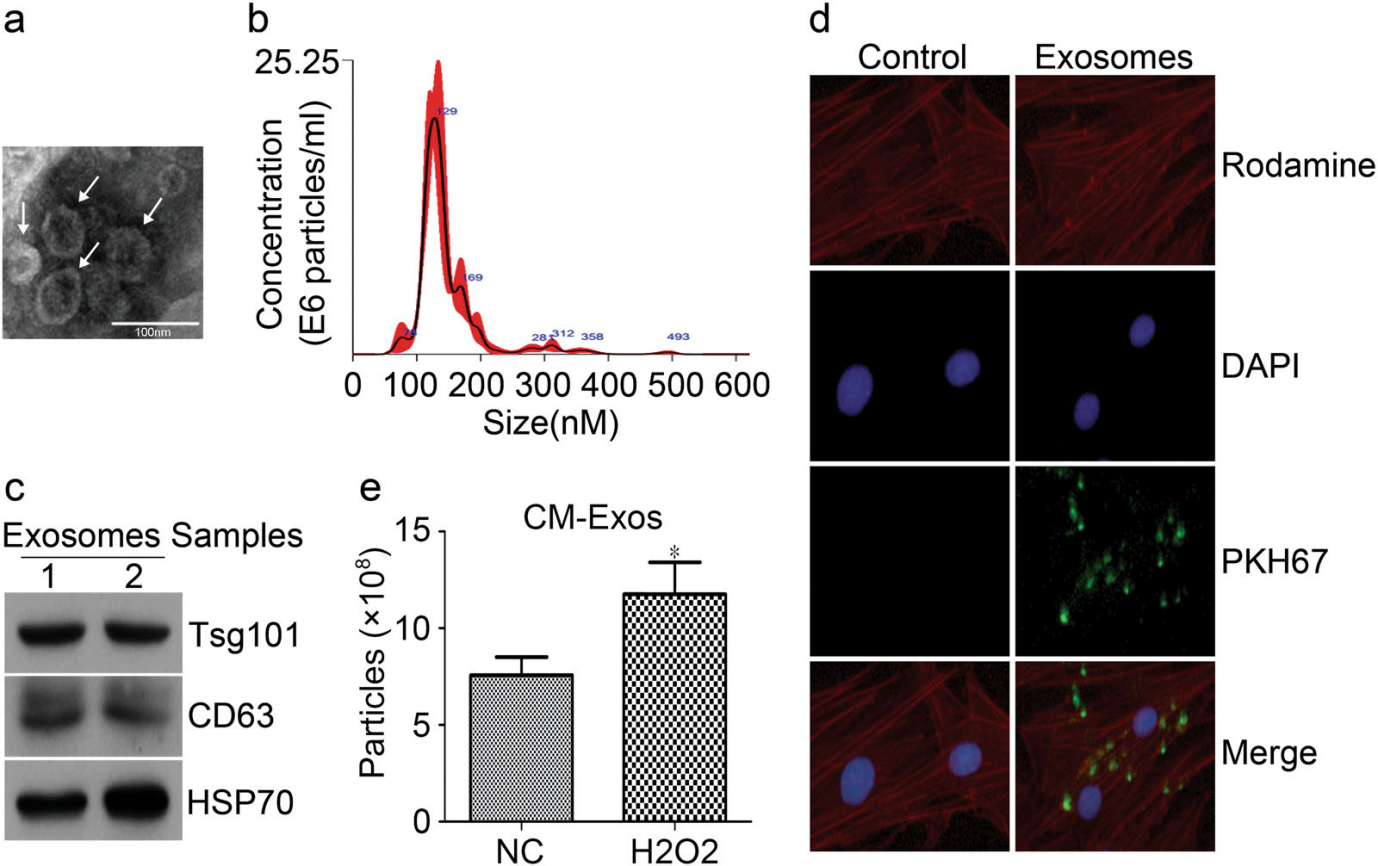
**Characteristics of cardiac exosomes.** **a** The size and morphology of exosomes were confirmed by transmission electron microscopy (TEM). The arrows indicated the typical exosomes. Scale bar, 100 nm. **b** The exosome size was measured using nanoparticle tracking analysis (NTA). The size of the exosomes between 100 and 200 nm, and the mode of these particles is 128.2 nm (*n* = 3 independent experiments). **c** Exosomal markers including CD63, Tsg101and Hsp70 were measured by western blot. **d** Uptake of cardiac exosomes by BMSCs. BMSCs were cultured with PKH67-labeled cardiac exosomes or control medium for 24 h and stained with DAPI and rhodaminephalloidin, the images were recorded under an inverted fluorescence microscopy. **e** The exosome concentrations between conditioned medium from CMs mixed with or without 100 μM H_2_O_2_ for 24 h were recorded by NTA. * *p* < 0.05 versus NC. All values were expressed as mean ± SD, *n* = 4 for each group

To obtain cardiac exosomes, the H_2_O_2_-cdM was collected and differentially ultracentrifuged. The identification of the morphology and properties of isolated particles was performed as previously described^17^. First, TEM demonstrated that the particles exhibited rounded and double-membrane structures with a size of ~50 nm (Fig. 3a). Secondly, nanoparticle tracking analysis (NTA) was performed to define the concentration and size distribution of the particles; a concentration of 1.07 × 10^9^ ± 1.43 × 10^8^ particles per ml was found with diameter between 100 and 200 nm, with the mode of 128.2 nm (Fig. 3b). Thirdly, Western blot was used to detect the exosomal protein markers CD63, HSP70, and Tsg101; all three markers were detected in the particles (Fig. 3c). Furthermore, the PKH67-labeled particles were cultured with BMSCs to confirm whether the secreted particles were incorporated into recipient BMSCs. Immunocytochemical analysis demonstrated that the PKH67-labeled particles (green fluorescence) were colocalized in the rhodaminephalloidin stained BMSCs (red fluorescence) after cultured for 24 h (Fig. 3d). Finally, the exosome concentrations between conditioned medium from CMs mixed with or without 100 μM H_2_O_2_ for 24 h were recorded by NTA. A total of 11.75 × 10^8^ ± 1.66 × 10^8^ particles per ml was found in H_2_O_2_ group and 7.57 × 10^8^ ± 0.94 × 10^8^ in NC group (Fig. 3e). So, the above results indicated that the CMs-derived particles collected in our experiments were exosomes, and that oxidative stress can promote the secretion of exosomes from CMs.

### Oxidative stressed cardiac exosomes promote H_2_O_2_-induced BMSCs apoptosis in vitro

**Fig. 4:**
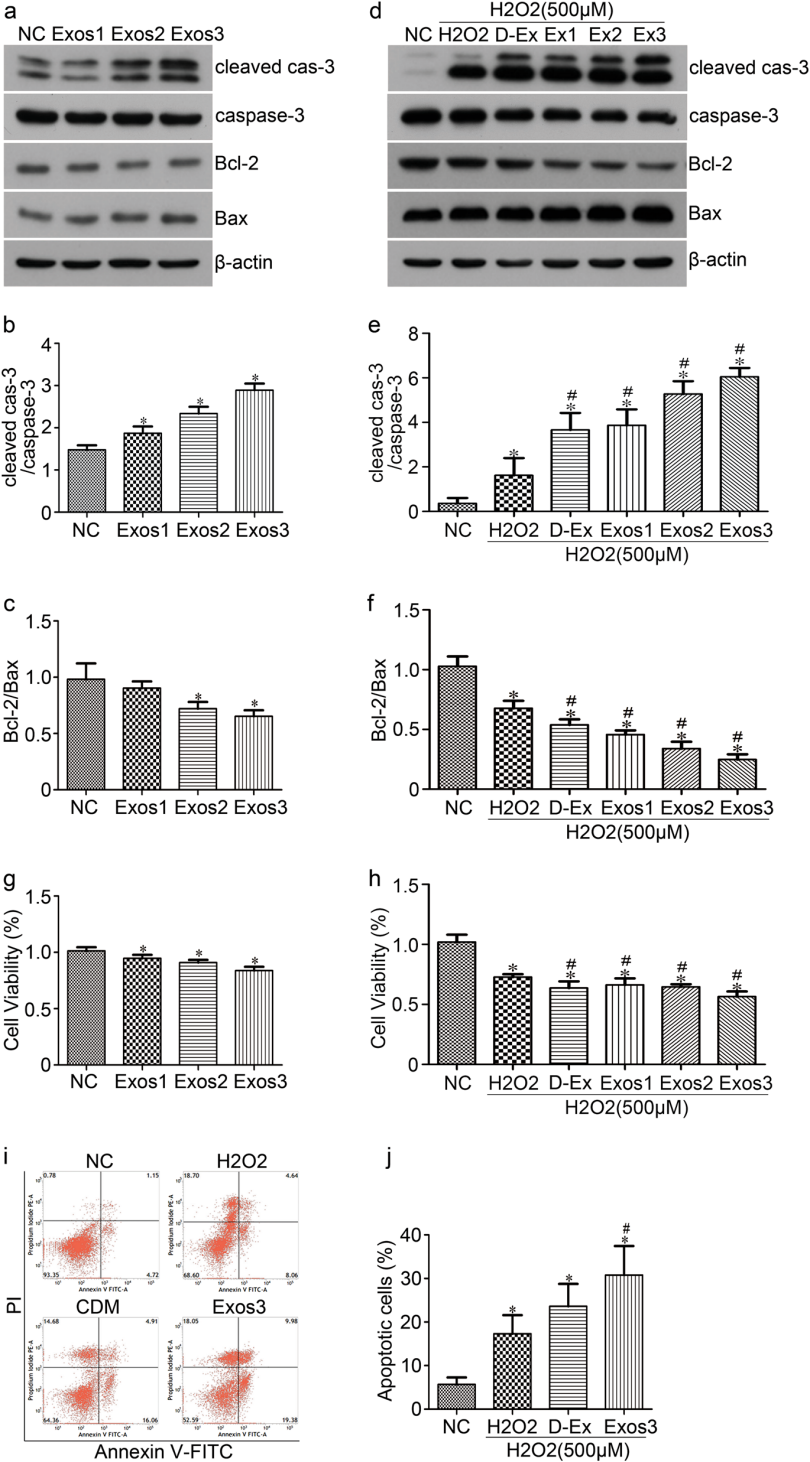
**The apoptotic effect of cardiac exosomes on BMSCs.** **a** BMSCs were lysated for detecting cleaved caspase-3, caspase-3, Bcl-2, Bax after 24 h culture with cardiac exosomes. Quantitative data of cleaved caspase-3/caspase-3 and Bcl-2/Bax ratio were shown (**b** and **c**). **d** BMSCs were lysated for detecting cleaved caspase-3, caspase-3, Bcl-2, Bax after 24 h pre-incubation with cardiac exosomes following treatment with serum-free DMEM and 500 μM H_2_O_2_ for additional 24 h. Quantitative data of cleaved caspase-3/caspase-3 and Bcl-2/Bax ratio were shown (**e** and **f**). **g** The cell viability of BMSCs after cultured with cardiac exosomes for 24 h was detected by CCK8 assay. **h** The cell viability of BMSCs after 24 h pre-incubation with cardiac exosomes and then treated with serum-free DMEM and 500 μM H_2_O_2_ for additional 24 h. **i** BMSCs cell apoptosis was assayed with Annexin V-FITC /PI dual staining by flow cytometry, representative flow images were shown. **j** The percentage of apoptotic BMSCs cells was summarized. *NC* BMSCs cells with DMEM containing 10% exosomes-depleted FBS, H_2_O_2_ BMSCs cells with 500 μM H_2_O_2_, D-Ex Exos-depleted H_2_O_2_-cdM, Exos Exosomes isolation from H_2_O_2_-cdM, Exos1 1 × 10^9^ particles/ml, Exos2 2 × 10^9^ particles/ml, Exos3 4 × 10^9^ particles/ml. *p < 0.05 versus NC, ^#^*p* < 0.05 versus H_2_O_2_. All values were expressed as mean ± SD, *n* = 3 for each group

Exosomes have an important role in mediating cell-to-cell communication. In this study, we explored the effect of cardiac exosomes on BMSCs apoptosis under normal culture or oxidative stress condition. As the previous results have shown that the effects of H_2_O_2_-cdM that promote H_2_O_2_-induced BMSCs apoptosis are more obvious than that of group cdM, we collected the conditioned culture medium of CMs in this condition and differentially ultracentrifuged isolate exosomes. BMSCs were cultured with 10% exosomes-depleted fetal bovine serum (FBS) DMEM containing three different concentrations of exosomes (1, 2, 4 × 10^9^ particles per ml) for 24 h in normal culture. Cell apoptosis was then determined by western blot; significant increase in cleaved caspase-3/caspase-3 and the decrease in Bcl-2/Bax ratio was observed, which were dependent on the exosomes concentration in normal culture (Fig. 4a–c). In addition, significant decreases in cell viability were observed in Exo1 (94.7 ± 3.1%) and Exo3 (83.9 ± 3.4%) group compared to NC group (101.3 ± 33.3%) (*p* < 0.05) (Fig. 4g).

To further explore the effect of cardiac exosomes on BMSCs apoptosis under oxidative stress condition, BMSCs were pre-incubated with 10% exosomes-depleted FBS DMEM with three different concentrations of cardiac exosomes for 24 h, and then treated with serum-free DMEM or exosomes-depleted conditioned medium (D-Ex) and with 500 μM H_2_O_2_ for additional 24 h. Subsequently, cell apoptosis was analyzed by western blot and Flow cytometry. A significant increase in cleaved caspase-3/caspase-3 and significantly decrease in Bcl-2/Bax ratio was found in D-Ex and Exos group; the level of cleaved caspase-3/caspase-3 was significantly increased and Bcl-2/Bax ratio was significantly decreased in Exos group compared with D-Ex (with the increased concentration of exosomes) (Fig. 4d–f). In addition, the cell viability in the Exo1 and Exo3 group decreased compared to H_2_O_2_ group (67.82% and 57.02% versus 73.23%, respectively) (Fig. 4h). Moreover, significantly increased cell apoptosis was found in the Exo1 group (30.79%) compared with the H_2_O_2_ group (17.31%) and D-Ex group (23.62%) (Fig. 4i, j). To sum up, these results suggested that oxidative stressed cardiac exosomes can promote BMSCs apoptosis.

### The identification of Rab27a KO mouse and the ability of exosomes secretion

**Fig. 5:**
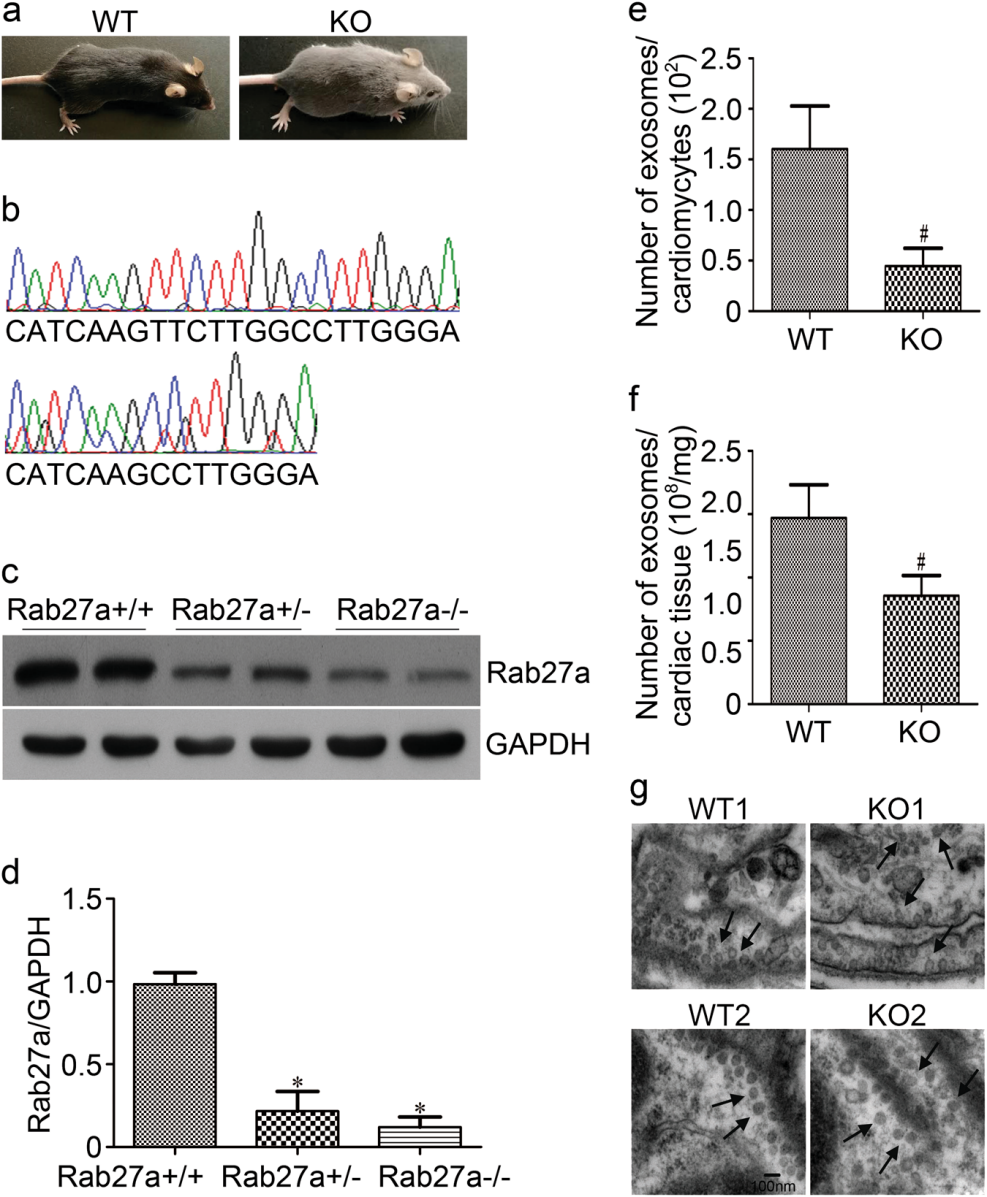
**The identification of Rab27a KO mouse and the ability of exosomes secretion.** Construction of Rab27a KO mice model by TALEN genome-editing technologies; the coat color phenotype of wild type (WT) and Rab27a KO mice (**a**), and the diagram of TALEN target sites (**b**). **c** The level of Rab27a protein in cardiac tissues of WT and KO mice were analyzed by western blot; GAPDH was employed as a loading control. Quantitative data of Rab27a band densities were shown (**d**). **e**,** f** Quantitative data of exosomes released from cardiomyocytes and cardiac tissues. **g** TEM micrograph of exosomes in cardiac tissues, the arrows indicated the typical exosomes. Scale bar, 100 nm. *p < 0.05 versus Rab27a+/+. ^#^*p* < 0.05 versus WT. All values expressed as mean ± SD, *n* = 4 for each group

In order to explore the role of cardiac exosomes in transplanted BMSCs in vivo, we constructed Rab27a KO mice model by designing and synthesizing highly active transcription activator-like effector nuclease (TALEN) specific to Rab27a exon 2 in the mouse genome (Fig. 5b), using a TALEN genome-editing technology. Rab27a, a member of Rab family of small GTPases, has a critical role in secretion of exosomes^15^. Previous studies have shown that Rab27a regulates melanosome transport in melanocytes. Dysfunction in this process has been proposed to cause type 2 Griscelli syndrome, a disease characterized by silvery hair^18^, which was also confirmed by our experiment (Fig. 5a), The Rab27a KO mouse had gray coat color compared to wild-type mouse. Rab27a knockout mice was confirmed by western blot; the level of Rab27a protein in cardiac tissues of Rab27a KO mice was notably downregulated compared to WT mice (*p* < 0.05) (Fig. 5c, d).

Consequently, several assays were performed to determine whether the exosomes released from cardiac cells of Rab27a KO mice were reduced. First, NTA was used to define the number of particles released from cardiac tissue and CMs. The exosomes were purified from the culture of CMs and cardiac tissues and then isolated using the previously described differential centrifugation protocols. NTA showed that the number of the secretory exosomes greatly decreased in KO CMs (44.7 ± 17.5/cell) compared to WT (160.3 ± 42.6/cell), and in KO cardiac tissues ((1.713 ± 0.320) × 10^8^/mg) compared to WT ((2.941 ± 0.525) × 10^8^/mg) (Fig. 5e, f). Secondly, TEM was used to observe the morphology of the exosomes directly. The particles exhibited round-shaped vesicles and diameter <100 nm, and the exosomes within the KO1 and KO2 cell cytoplasm were significantly increased compared to WT1 and WT2 group (the black arrows point), respectively (Fig. 5g), which suggested that exosomes released from Rab27a KO cells were reduced.

### The transplanted BMSCs survival for the therapy of MI in Rab27a KO mice were increased

**Fig. 6:**
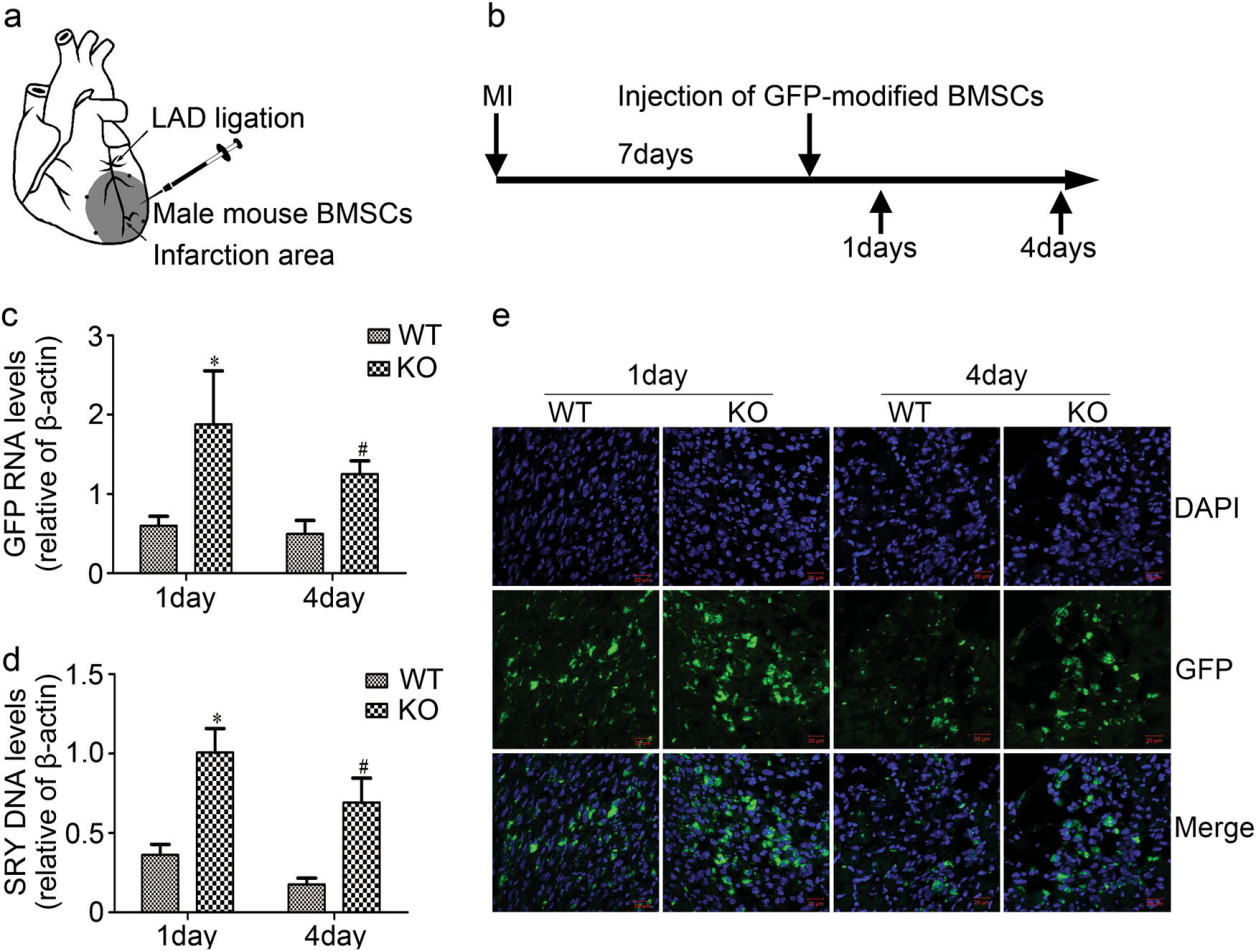
**Effects of transplanted BMSCs survival for treating myocardial infarction in Rab27a KO mice.** **a** Diagram of BMSCs therapy for myocardial infarction in vivo. After left anterior descending artery ligation, male mouse BMSCs were injected into 4 spots of border zone surrounding the infarcted zone of the female mouse heart. **b** Schematic depiction of BMSC transplantation and sample collection. **c**, **d** Real-time polymerase chain reaction analyzed Sry gene and GFP mRNA after 1 and 4 days of injection of BMSCs. *p < 0.05 versus 1d WT, #*p* < 0.05 versus 4 days WT. All values were expressed as mean ± SD, *n* = 5 for each group. **e** GFP fluorescence signal intensity (green fluorescence) and DAPI staining (blue fluorescence) of BMSCs in cardiac tissue were measured by confocal laser scanning microscopy. WT wild-type mice, KO Rab27a knockout mice. Scale bar, 20 μm

To further validate the role of cardiac exosomes on the transplanted BMSCs survival in vivo, we transplanted 2.0 × 10^6^ male mouse GFP-modified BMSCs into the viable myocardium bordering the infarction in Rab27a KO and wild-type female mice (Fig. 6a, b). RT-PCR for mice Y-chromosome Sry DNA and GFP mRNA were used to estimate the survival of implanted cells. Our results showed that the transplanted BMSCs survival increased in Rab27a KO mice by the higher level of Y-chromosome Sry DNA and GFP mRNA, with approximately three-fold enhancement by day 1 and day 4 post cell transplantation (*n* = 5, *p* < 0.05) (Fig. 6c, d). We also used confocal laser scanning microscopy to analyze the GFP fluorescence signal intensity of transplanted cells in heart tissues. The results were consistent with the results of real-time PCR; stronger GFP signal was found in KO group compared to WT group at day 1 and day 4, respectively (Fig. 6e). These data indicated that the transplanted BMSCs survival in infarcted heart was increased in Rab27a KO mice, suggesting that cardiac exosomes may accelerate transplanted BMSCs injury after MI.

## Discussion

There are ~1,000 million CMs lost during MI, while typical dose of transplanted cells can reach or even exceed this number. However, only few of the cells may successfully engraft into the myocardium, and are much less likely to continuously survive after transplantation^19,20^. Much of this attrition can be attributed to the harsh microenvironment in and near the region of the infarct; nonetheless the exact mechanism is still not fully understood^21^. To explore whether the injured CMs have an ability to affect the survival of transplanted cells by paracrine action, we cultured BMSCs with CMs conditioned medium under oxidative stress in vitro. Western blot revealed that the expression of apoptosis-related proteins was significantly increased and cell viability was decreased in group cdM and group H_2_O_2_-cdM compared to their respective control. Also, the cell injury in group H_2_O_2_-cdM was more worse compared to group cdM. These findings demonstrated that CMs conditioned medium could promote H_2_O_2_-induced BMSCs apoptosis; group H_2_O_2_-cdM showed more obvious effects compared to group cdM. Accordingly, we explored what substances in the group H_2_O_2_-cdM take effects.

Cellular cross-talk has a central role for adaptive response of organisms to stress and maintain homeostasis^22^. Direct contact and secreted molecules, including cytokines, growth factors, and secreted exosomes, are two major manners for cellular interaction^23^. Recently, studies have found that exosomes produced by CMs regulate endothelial cell (EC), cardiac fibroblast, and neighboring CMs^24–26^. We explored whether oxidative stressed cardiac exosomes have effect on BMSCs apoptosis under normal culture or oxidative stress. Apoptosis is an automatic and programmed process mediated by the intracellular proteolytic cascade, which includes the extrinsic pathway and the intrinsic (or mitochondrial) pathway. The two types of caspases are initiator caspases (such as caspase-8 and 9) and executioner caspases (caspase-3, 6, and 7). Activated initiator caspases cleave executioner caspases (such as caspases-3 to cleaved caspase-3, the active form of caspase-3), which execute apoptosis^27,28^. Activation of the intrinsic pathway is mainly mediated by the Bcl-2 family, among which the pro- and anti-apoptotic molecules (Bax and Bcl-2) are the most common markers for apoptosis^29^. The obtained results showed that the expression of apoptosis-related proteins and apoptotic rates were greatly increased in group D-Ex and Exos, while the increased levels were significantly higher in group Exos (with the increased concentration of exosomes). This suggested that the exosomes in oxidative stressed CMs conditioned medium may have an important role in promoting H_2_O_2_-induced BMSCs apoptosis.

There are various cell types in the heart: CMs, fibroblasts, CPCs, ECs, and vascular smooth muscle cells (VSMCs). Several studies have demonstrated that CMs, ECs, fibroblasts, and CPCs can produce and secret exosomes so as to regulate the function of heart in specific condition^30–33^. Secreted exosomes can transport nucleic acids, lipids, and proteins via interstitial fluid and circulating blood, and thus can be involved in local and remote intercellular communication^34,35^. Since we already showed that oxidative stressed cardiac exosomes promote H_2_O_2_-induced BMSCs apoptosis, we wanted to further elucidate whether exosomes secreted from cardiac fibroblasts, ECs, or VSMCs work in the same way.

Cardiomycytes did not act as typical secretory cells. However, in 2007, Gupta and Knowlton^36^ were first who isolated exosomes released from adult rat CMs by using differential ultracentrifugation techniques. To gain insight into the function of exosomes, the exosome proteomics of cardiac cells were also analyzed^37^. In contrast to other EVs, cardiac EVs have a specific signature of the proteome, which highlights its origins in the heart muscle. Moreover, the inducible secretion of exosomes protein is regulated by pathological changes in the environment, including fever, hypoxia, and oxidative stress. Unlike Hsp70 and Hsp90, the heat shock protein 60 (Hsp60) contained in the cardiac EVs has not been previously found in other cell types derived EVs. It has been suggested that extracellular HSP60, when not in exosomes, may trigger cardiomyocyte apoptosis and inflammation through a Toll-like receptor (TLR)-mediated mechanism^38,39^. Tumor necrosis factor-α (TNF-α) is a cytokine that regulates multiple biological processes in human. TNF-α is released soon after myocardial ischemic injury, and it contributes to myocardial injury and dysfunction^40^. Under hypoxia, CMs are induced by hypoxia inducible factor 1α (HIF-1α) to generate more TNF-α, which are encapsulated by exosomes and then released. Incubating these TNF-α containing vesicles with CMs increases apoptosis in CMs^41^. These studies have shown that CMs have the ability to transport protein to other cells by generating and secreting exosomes so as to regulate cell survival or apoptosis. These findings instigated us to further explore the mechanism underlying cardiac exosomes-accelerated transplanted BMSCs injury.

In conclusion, this study demonstrated that cardiac exosomes accelerate transplanted BMSCs injury in infarcted heart, revealing a previously unrecognized mechanism underlying the poor survival of transplanted cells. Moreover, blocking the release of cardiac exosomes or its detrimental contents could be used as an effective strategy to enhance implanted BMSCs survival after MI.

## Materials and Methods

### Materials

Hydrogen peroxide (H_2_O_2_) was manufactured by Sigma-Adrich Inc. (St. Louis, MO, USA). Antibodies raised against β-actin, Bax, cleaved caspase-3, caspase-3, Tsg101, HSP70, and GAPDH were obtained from Cell Signaling Technology (Beverly, MA, USA) and anti-Bcl-2, CD63 were purchased from Bioworld Technology, Inc. (USA). Enhanced chemiluminescence (ECL) kit was provided by Santa Cruz Biotechnology Inc.

### Animals

Animals were raised and treated in accordance with the Guangdong Animal Center for the ethical treatment of animals. We claim that the animal experiments in this study were approved by the Institutional Animal Care and Use Committee of Guangzhou Medical University (Guangzhou, China). A total of 160–180 g adult male Sprague-Dawley rats and 15–18 g C57BL/6 mice were obtained from Guangdong Laboratory Animal Monitoring Institute. During the study, the animals had access to standard laboratory diet and drinking water adlibitum.

### Purification of BMSCs and neonatal rat CMs

Primary isolation of rat and male mouse BMSCs were performed according to previously established methods^42^. The BMSCs were cultured with DMEM (Gibco) containing 10% (FBS) and antibiotics (Gibco). Culture medium was replaced every 3 days and the removal of nonadherent hematopoietic cells was performed. Passage 3 BMSCs were used for further experiments.

Isolation of neonatal rat CMs was performed as previously described^43^. Briefly, left ventricles from 1 to 2-day old rats were collected and sliced into small pieces, and then digested with collagenase Type II (Gibco) at 37 °C. After digestion, the cells were resuspended in DMEM containing 10% FBS. Then, differential preplating was performed to enrich CMs, which were seeded in 10%FBS DMEM containing 0.1 mM bromodeoxyuridine (BrdU). Culture media were changed after 48 h incubation and the cells were used for further experiments.

### In vitro model of oxidative stress injury

To mimic the oxidative stress microenvironment after MI that the CMs or transplanted BMSCs encounter in vivo, the cells were exposed to different concentrations of hydrogen peroxide (H_2_O_2_) in serum-free DMEM (Gibco) for 24 h. The cell viability was detected by the Cell Counting kit-8 (CCK-8; Dojindo Laboratories) according to the manufacturer’s instructions. For the detection of the cleaved caspase-3 and caspase-3, protein lysates were prepared and detected by western blot analysis.

### In vitro experiments with conditioned medium

Conditioned medium was prepared as follows: 80–90% confluent CMs were cultured with serum-free DMEM (cdM) or serum-free DMEM and subjected to 100 μM H_2_O_2_ treatment (H_2_O_2_-cdM) for 24 h. Ctr-cdM (without CMs) and Ctr-H_2_O_2_-cdM (without CMs) served as control of cdM and H_2_O_2_-cdM, respectively. The collected culture supernatant was centrifuged at 1,500 rpm for 10 min, and was used immediately or after being stored at −20 °C. After the 80–90% confluence, BMSCs were cultured with controlled medium (Ctr-cdM and Ctr-H_2_O_2_-cdM) or conditioned medium (cdM and H_2_O_2_-cdM) and then mixed with or without 500 μM H_2_O_2_ for 24 h. Cell viability analysis was detected using CCK-8. Cell apoptosis was determined by western blot.

### Isolation and characterization of exosomes

The cardiac exosomes isolation procedures were performed by differential ultracentrifugation^44^. Briefly, 50 ml serum-free DMEM was used for culturing CMs in two T175 flasks. After treatment with 100 μM H_2_O_2_ for 24 h, supernatant was differentially centrifuged at 300x *g* for 10 min, 2000x *g* for 10 min and 10,000x *g* for 60 min to remove any residual cells and debris, then centrifuged at 120,000x *g* for 90 min at 4℃ to precipitate exosomes. The medium that removed exosomes after centrifugation served as exosomes-depleted conditioned medium (D-Ex) for culturing with BMSCs. The isolated exosomal precipitates were washed once with cold sterile phosphate-buffered saline (PBS), then suspended in 50 μl of PBS or dissolved in lysis buffer for protein extraction.

The concentration and size distribution of exosomes were confirmed by NTA using NanoSight NS300 (Malvern, UK). The differences in exosome concentrations between conditioned medium from CMs mixed with or without 100 μM H_2_O_2_ for 24 h were recorded. Three recordings were performed for each sample. Western blot was used to detect the exosomal protein markers (CD63, HSP70, and Tsg101).

For the Transmission Electron Microscopy (TEM) morphology observation, 3 μl of exosome suspension was absorbed onto formvar carbon-coated copper electron microscopy grids (200 mesh) at room temperature for 5 min, and then subjected to 2% uranyl acetate staining for an additional minute. Grids were washed three times with PBS and were maintained in semi-dry state before observation by TEM (Hitachi H7650 TEM, Japan).

### Exosome Uptake assays

The PKH67 green fluorescent cell linker kit was used for labeling exosomes according to the manufacturer’s instructions (Sigma Aldrich). Briefly, exosomes resuspended in buffer were mixed with PKH dyes at room temperature for 5 min. Consequently, the pellets were added to PBS containing 5% bovine serum albumin (BSA) and subjected to ultracentrifugation at 120,000x *g* for 90 min to remove free dye. The samples were then resuspended in 50 μl DMEM containing 10% exosomes-depleted FBS. Labeled exosomes were cultured with BMSCs for 24 h. Finally, exosome-PKH67-treated BMSCs were fixed and stained with rhodaminephalloidin and DAPI, and evaluated by fluorescence microscopy.

### BMSCs cultured with oxidative stressed cardiac exosomes

BMSCs were cultured with three different concentrations of exosomes (1, 2, 4 × 10^9^ particles per ml) for 24 h in normal culture (DMEM containing 10% exosomes-depleted FBS). In exploring the effects of cardiac exosomes on BMSCs apoptosis under oxidative stress, BMSCs were pre-incubated with three different concentrations of exosomes (as previous described) for 24 h, and then treated with serum-free DMEM and 500 μM H_2_O_2_ for 24 h. Following treatment, western blot, and Flow cytometry with Annexin V-FITC/PI kit (eBioscience) were used to analyze the cell apoptosis, while CCK-8 assay was used to assess cell viability.

### Cell apoptosis analysis by Flow cytometry

Cell apoptosis was analyzed using Annexin V-FITC Apoptosis Detection Kit (eBioscience), following the manufacturer’s instructions. Briefly, the cells were collected and washed with PBS and then resuspended in 200 μl binding buffer. Consequently, cells were mixed with 5 μl Annexin V-FITC at room temperature for 10 min following the incubation with 10 ul propidiumIodide (PI). Finally, early and late apoptosis were analyzed using FACS cytometry (BD FACSVerse).

### Western blot

Western blot analysis was performed as previously described^45^. Briefly, proteins were collected, normalized, and separated by 12% SDS-PAGE and electrotransferred to methanol-treated polyvinylidene difluoride (PVDF) membranes. 5% nonfat milk in TBST for 1 h at room temperature was used to block the membranes. The membranes were then incubated with antibodies overnight at 4 °C, followed by HRP linked secondary antibody. The detection was performed by the ECL system.

### Constructing Rab27a KO mice model by a TALEN genome-editing technique

TALENs are a combination of the FokI endonuclease and the TAL effector DNA-binding domain, which can be engineered to cut specific sequences of DNA^46^. For constructing Rab27a KO mice model, we designed and synthesized highly active TALEN specific to exon 2 of Rab27a. The engineered restriction enzymes were then introduced into cells, for genome editing of Rab27a in situ. Coat color phenotype and the protein expression of Rab27a were analyzed to identify Rab27a KO mice.

### The identification of the ability of exosomes secretion in Rab27a KO mouse

The exosomes released from neonatal mouse CMs were isolated using the same method as for rat CMs. The exosomes released from cardiac tissue were isolated using a previously described method^47^. Briefly, the heart homogenate was sequentially filtered through a 40 μm mesh filter (BD) and a 0.2 μm syringe filter (Thermo). The filtrate was then condensed through a 100 kD ultrafiltration centrifuge tube (Amicon ultra 15; MiIlipore). The exosomes from the condensed filtrates were isolated using the differential ultracentrifugation protocols. The concentrations of cardiac exosomes were confirmed by NTA, and TEM micrograph was used for analyzing exosomes distribution in cardiac tissues.

### Induction of MI and BMSCs Transplantation

A MI model was constructed in female mice (20–23 g). Briefly, under spontaneous isoflurane inhalation induced anesthesia, the animals underwent tracheal intubation via oral cavity and were mechanically ventilated with room air supplemented with oxygen using a rodent ventilator (ALCBIO-V8S). The heart was exposed by left-side thoracotomy and the left anterior descending artery (LAD) was ligated 2 mm from its origin between the pulmonary artery conus and the left atrium using 9–0 polyester suture. Successful infarction was confirmed by the blanching of the left ventricular muscle and an ST elevation on electrocardiograms.

Passage 3 male mouse BMSCs were transduced with an adenoviral vector encoding the GFP reporter gene (vigene). Transduction efficiency was evaluated by detection of GFP fluorescence signal intensity. GFP-modified BMSCs were detached with 0.125% (w/v) trypsin, suspended in PBS, and kept on ice until injection. Seven days after coronary ligation, the surviving mice received re-thoracotomy and an intramyocardial injection of 2.0 × 10^6^ cells (total of 50 ul in PBS) with a 30-gauge needle; the injection was performed at four sites into anterior and lateral aspects of the viable myocardium bordering the infarction. Two groups (20 mice/group) were used in this study, including wild-type mice (WT) and Rab27a KO mice (KO). Each group was then divided into two additional subgroups (10 mice/group). One group was subjected to real-time PCR for determining the survival rate after 1and 4 days of cell transplantation. The other group underwent for confocal laser scanning microscopy analysis.

### Real-Time PCR for implanted BMSCs Survival

The mouse heart samples from different groups were electrically-driven tissue homogenated and the genomic DNA and total RNA were isolated using TRIzol Reagent (Invitrogen). The concentration and purity of the DNA or RNA were measured by spectrophotometry. GFP-cDNA was reverse-transcripted using ReverTra Ace qPCR RT Kit (TOYOBO). Real-Time PCR was performed for quantification of the Sry-DNA and GFP-cDNA of heart tissues after 1- and 4-days of cell implantation with THUNDERBIRD SYBR qPCR MiX Kit (TOYOBO).The primer sequences for amplification of GFP, mouse Y-chromosome Sry, and β-actin genes are listed below: GFP: sense, 5′-AAGTTCATCTGCACCACCG-3′; antisense, 5′-TCCTTGAAGAAAGGTGCG-3′; Sry: sense, 5′-CTGCTGTGAACAGACACTAC-3′; antisense, 5′-GACTCCTCTGACTTCACTTG-3′; β-actin: sense, 5′-TGGCTCCTAGCACCATGAAG-3′; and antisense, 5′-AACGCAGCTCAGTAACAGTCC-3′. The cycling conditions were set at 2 min at 95 °C for initial prenaturation, 40 cycles of denaturation at 95 °C for 5 s, annealing at 60 °C for 15 s, and extension at 72 °C for 60 s. The PCR-amplified DNA was quantified and the results were normalized against β-actin expression. The relative PCR products were calculated with the 2^−ΔΔCt^ method.

### Confocal laser scanning microscopy

For detecting the survival rate of transplanted BMSCs, the GFP fluorescence signal intensity of transplanted cells in heart tissues were examined with confocal laser scanning microscopy as previously described^48,49^. Briefly, heart tissues after 1- and 4- days of cell implantation were made into frozen sections, then fixed in 3.7% buffered paraformaldehyde for 10 min. Staining of 40, 6-diamino-2-phenylindole (DAPI) (sigma-Aldrich) indicated the nucleus. Finally, the GFP fluorescence signal intensity of transplanted cells in heart tissues was examined with confocal laser scanning microscopy.

### Statistical analyses

Data in this study are expressed as the means ± standard deviation (SD). Differences were compared by analysis of variance (ANOVA) or *t-*test, as appropriate. A value of *p* < 0.05 was considered statistically significant. SPSS 13.0 was used to analyze the data.
